# Analysis of justification for author order and gender bias in author order among those contributing equally

**DOI:** 10.1128/mbio.00646-24

**Published:** 2024-03-29

**Authors:** Ellie Rose Mattoon, Maisha Miles, Nichole A. Broderick, Arturo Casadevall

**Affiliations:** 1 Department of Molecular Microbiology and Immunology, Johns Hopkins Bloomberg School of Public Health, Baltimore, Maryland, USA; 2 American Society for Microbiology, Washington, DC, USA; 3 Department of Biology, Johns Hopkins University, Baltimore, Maryland, USA; Institut Pasteur, Paris, France

**Keywords:** gender bias, academia, diversity, equity, inclusion, equal contribution, research, gender

## Abstract

The practice of designating two or more authors as equal contributors (ECs) on a scientific publication is increasingly common as a form of sharing credit. However, EC authors are often unclearly attributed on curriculum vitae (CVs) or citation engines, and it is unclear how research teams determine author order within an EC listing. In response to studies showing that male authors were more likely to be placed first in an EC listing, the American Society for Microbiology (ASM) required that authors explain the reasons for author order beginning in 2020. In this study, we analyze data from over 2,500 ASM publications to see how this policy affected gender bias and how research teams are making decisions on author order. Data on publications from 2018 to 2021 show that gender bias was largely nonsignificant both before and after authors were asked by ASM to provide an EC statement. The most likely reasons for EC order included alphabetical order, seniority, and chance, although there were differences for publications from different geographic regions. However, many research teams used unique methods in order selection, highlighting the importance of EC statements to provide clarity for readers, funding agencies, and tenure committees.

**IMPORTANCE:**

First-author publications are important for early career scientists to secure funding and educational opportunities. However, an analysis published in *eLife* in 2019 noted that female authors are more likely to be placed second even when both authors report they have contributed equally. American Society for Microbiology announced in response that they would require submissions to include a written justification of author order. In this paper, we analyze the resultant data and show that laboratories are most likely to use some combination of alphabetical order, seniority, and chance to determine author order. However, the prevalence of these methods varies based on the research team's geographic location. These findings highlight the importance of equal contributor statements to provide clarity for readers, funding agencies, and tenure committees. Furthermore, this work is critically important for understanding how these decisions are made and provides a glimpse of the sociology of science.

## INTRODUCTION

The number of scientific journal articles listing two or more authors as equal contributors (ECs) has steadily increased since the 1990s (1). This trend occurs concurrently with increases in the total number of authors on a publication (2) and increases in international collaboration (3). Because funding, tenure, and hiring committees often use an author’s byline position as a metric of relative contribution, EC presents a potential solution to ensure that collaborators receive the appropriate credit for their contributions (4).

Despite these efforts, EC authors still receive less credit if their name is listed second on the byline. Readers instinctively associate the first position with greater influence, prestige, and importance based on the long tradition of recognizing the first author as the major contributor to published works in the biomedical sciences. This problem of misperception is accentuated by the fact that EC is often not acknowledged on reference lists or automated research profiles (4), and it is currently unclear how EC is viewed in hiring and promotion decisions (1). A 2017 survey of 6,000 corresponding authors found that contribution statements were more likely to be used to assess a type of contribution rather than effort or credit (5). In addition, given the diversity of social dynamics on research teams and laboratories, the methods used to determine author order even in cases of EC vary widely from paper to paper (5).

In 2019, an analysis of 2,898 scientific papers with listed EC authors found that a male author was more likely to be listed first than a female counterpart on an EC publication (6), although the phenomenon was declining over time. A similar analysis of 10 journals with high-impact factors found that although there was an insignificant gender bias in basic science journals, male authors were more likely to be listed first in clinical journals (7). Women are already less likely to be credited with authorship on a project (8), and although women receive 41% of science, technology, engineering, and mathematics (STEM) doctorate degrees, they hold only 26% of STEM-related leadership roles (9). This imbalance in first-author contributions parallels imbalances in citations, funding, and other forms for recognition for women scientists. Our prior findings about EC authorship led to wider conversations on how EC practices might reflect or perpetuate gender inequalities in science (10).

In response to these studies, the *Journal of Clinical Investigation* (JCI) (10) and journals of the American Society for Microbiology (ASM) (11) announced that they would be requiring corresponding authors to state the method used in assigning first-author position. In a statement, JCI leadership announced that they aimed to promote “discussions between authors and their supervisors that could lead to fairer choices” (10). There was also hope that by requiring explanations for the author’s order that information could help those listed second to explain the decision and obtain their fair share of credit (11). In this study, we analyze the outcome of the policy requiring author order explanations among those contributing equally. We found a wide range of methods used to order EC authors, showcasing the importance of reviewing EC publications and author contributions on a case-by-case basis.

## MATERIALS AND METHODS

### Data sources

All analyzed data were taken from the submission information from 3,177 publications from American Society for Microbiology journals published between 2018 and 2021, all of which specified two equal contributors in either the first, middle, or final position on their submission information. Starting in 2020, submissions also were asked to include an explanation for how author order was determined among equal contributors.

### Data analysis

Methods are replicated from those used in reference 6. One of the coauthors manually examined each article to determine the gender of each provided author. Determination of an author’s presenting gender was primarily completed by searching an individual’s full name and affiliated institution on the Google search engine. Oftentimes, the image and pronouns on their faculty or student profile were sufficient to determine the individual’s gender presentation. For individuals who did not have an identifying photo or who had a name commonly perceived as gender-neutral (such as “Avery” or “Leslie”), that paper’s corresponding author was emailed and asked if they could provide their colleague’s gender identity. A portion of corresponding authors either did not respond after three contact attempts or asked to opt out of the study. This study was unable to account for nonbinary individuals in its analysis.

For each entry, we also recorded the byline position of the ECs (first vs last), the country of the corresponding author, and the method of author order by the following categories: alphabetical, random, and other. After completing the initial analysis, an additional category, “seniority,” was added to account for the large number of entries claiming to use this method.

### Statistics

Gender bias in authorship was defined in two ways: the proportion of EC papers with two authors of different genders that presented author bylines as male-female and the proportion of all EC papers that had a male in the first-author spot (this includes male-female [M:F] entries, male-male [M:M] entries, and entries with three or more ECs in which a male is listed first [M+]). For both pairings, a proportion closer to 50% was assumed to have less gender bias.

For the regional analysis of provided entries, each entry was categorized into the following world regions: North America, Europe, Asia, South America, the Middle East, Africa, and Australia. Due to the low sample size, South America, the Middle East, Africa, and Australia entries were either excluded from some analyses or combined into an “other” category. Entries with corresponding authors from multiple continents were excluded from the analysis of regional characteristics of author order methods.

All data analysis was carried out using Excel.

## RESULTS

### Summary data among ASM journals

From 3,172 scientific publications in 13 ASM journals published between 2019 and 2021, 2,665 (84%) usable entries (entries for which we could obtain gender information) were identified (Table 1). Of the provided entries, 16% were excluded after the research team was unable to identify the ECs’ genders, and the corresponding author either failed to respond to email inquiries or asked to opt out of the analysis. Of the 3,172 entries, 65% were successfully identified using internet resources alone, and the success rate of email inquiries was 54% (Table 2).

**TABLE 1:** Summary data of ASM authors listed as equal contributors, organized by journal and author gender

			Contributed equally = 2				Contributed equally > 2	
Journal title	Total entries	Usable entries	M:M*^a^*	M:F^*a*^	F:F^*a*^	F:M^*a*^	M+^*b*^	F+^*b*^
*Antimicrobial Agents and Chemotherapy*	417	355	80	77	77	74	25	31
*Applied and Environmental Microbiology*	341	272	67	52	73	55	17	14
*Clinical Microbiology Reviews*	14	12	4	3	3	0	1	1
*Infection and Immunity*	162	136	33	26	42	27	7	5
*Journal of Bacteriology*	120	112	34	20	26	21	6	6
*Journal of Clinica****l*** *Microbiology*	215	187	53	40	40	38	17	8
*Journal of Virology*	638	490	109	103	93	106	46	50
*mBio*	628	582	160	96	105	116	68	50
*mSphere*	164	139	37	28	31	26	12	12
*mSystems*	156	123	40	20	19	25	15	7
*Microbiology and Molecular Biology Reviews*	19	16	2	5	4	4	1	0
*Microbiology Resource Announcement*	229	181	46	36	34	39	13	13
*Molecular and Cellular Biology*	69	60	13	8	12	15	7	7
	3,172	2,665	678	514	559	546	235	204

^*a*^ M:M, M:F, F:M, and F:F represent papers in which two authors share a position, with the order of authors being male-male, male-female, female-male, and female-female, respectively.

^*b*^ For papers with three or more authors sharing an EC position, “M+” means that the first listed author is a male, and “F+” means that the first listed author is a female.

**TABLE 2:** Summary data of methods used to identify author gender (internet vs email) and email inquiry success rate, organized by year of journal publication

Year	Internet	Emails (total)	Successful emails	Excluded
2019	684	385	197	188
2020	753	439	233	206
2021	627	284	170	114
Total	2,064	1,108	600	508
Proportion	0.65	0.35	0.19	0.16

### The impact of EC justification statement on gender bias

ASM began requiring EC justification statements for journals published in 2020. One entry from 2019 provided that the authors were ordered alphabetically without being asked to (Table 3). Approximately 9% of entries from 2020 and 2021 explained that their EC authors contributed equally, but they failed to add any supplementary information as to why a given author was added first, despite the journal requirements, which could reflect inadequate enforcement.

**TABLE 3:** EC author gender, byline order, and method of determining first author organized by year^*c*^

Year	M:M^*a*^	M:F^*a*^	F:M^*a*^	F:F^*a*^	M+^*b*^	F+^*b*^	First	Last	Alphabetical	Other	Random	Seniority
2019	229	159	192	184	69	72	745	124	1	0	0	0
2020	251	193	203	209	83	76	836	150	253	338	62	228
2021	198	162	151	166	83	56	656	124	185	276	68	160
Sum	678	514	546	559	235	204	2,237	398	439	614	130	388

^*a*^ M:M, M:F, F:M, and F:F represent papers in which two authors share a position, with the order of authors being male-male, male-female, female-male, and female-female, respectively.

^*b*^ For papers with three or more authors sharing an EC position, “M+ means that the first listed author is a male, and “F+” means that the first listed author is a female.

^*c*^ Submissions in 2019 were not asked to provide reasons for why a given author was included first on the byline.

Other factors are important to note when examining the tables below . Several 2020 and 2021 statements gave multiple reasons for author order (for example, alphabetical order and seniority) and thus are coded into multiple categories (Table 3). Some entries provided multiple EC statements for both the first and last author positions and are coded into both the “first” and “last” categories, while other entries only had EC authors in a middle byline position and thus are coded into neither category (Table 3).

**Fig 1:**
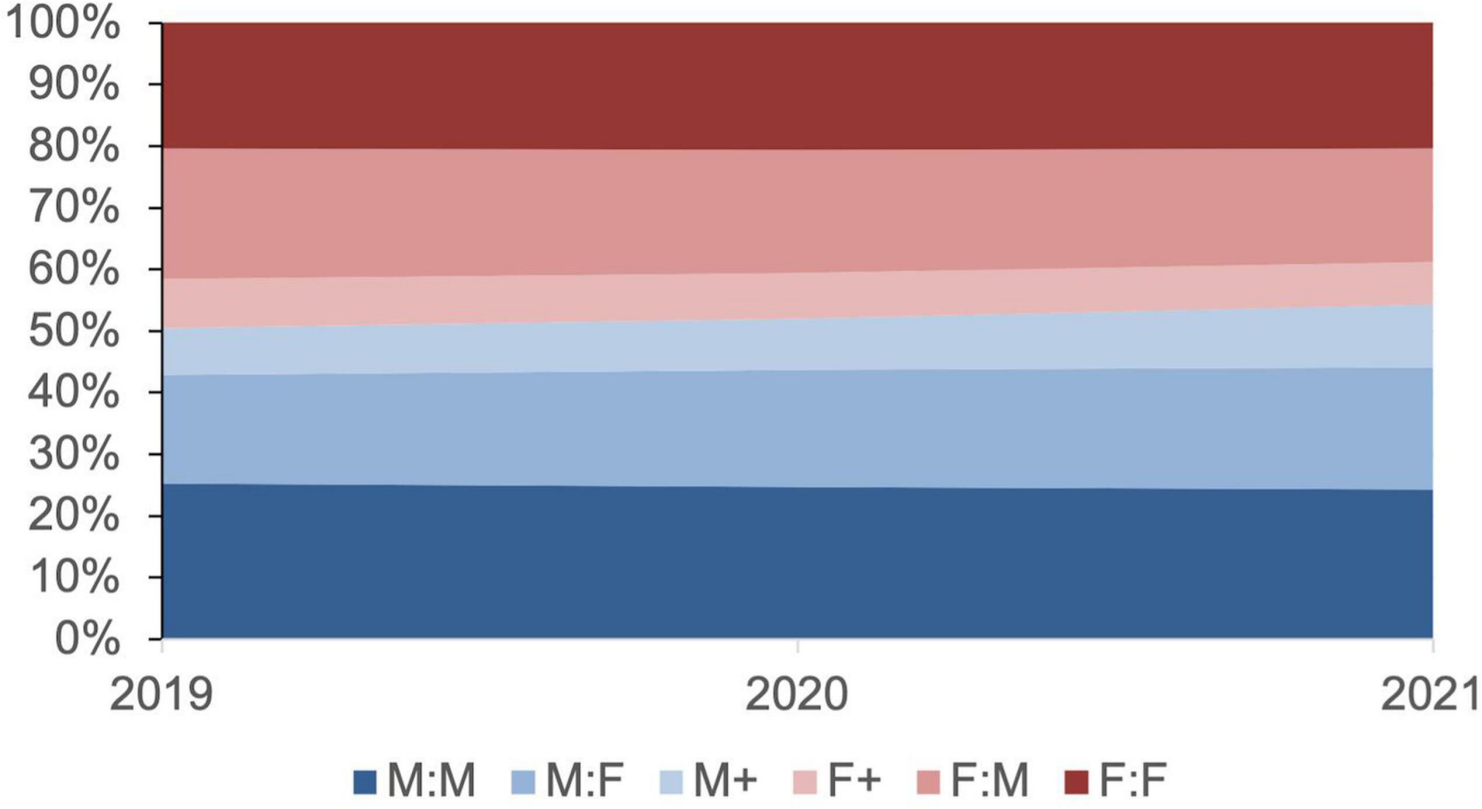
Proportion of gender combinations among EC authors in ASM journals between 2019 and 2021. M:M, M:F, F:M, and F:F represent papers in which two authors share a position, with the order of authors being male-male, male-female, female-male, and female-female, respectively. For papers with three or more authors sharing an EC position, “M+“ means that the first listed author is a male, and “F+” means that the first listed author is a female. The proportion of combinations in which a male author is listed first is shown in various shades of blue, while female-first combinations are shown in shades of red.

When defining gender bias as any male author placed first, there was a nonsignificant difference between entries submitted before and after ASM’s policy change (*P* = 0.23). This pattern continued when looking only at M:F papers in contrast to F:M papers (*P* = 0.16). Previous research has suggested that the phenomenon of listing male authors first had been decreasing over time (6), with submissions with a male first author approaching 50% in 2019 (Fig. 1). However, by 2021 male-first entries approached 54%. This increase did not achieve significance at the 0.05 level (*χ*^2^, *P* = 0.12), but the finding suggests the need for future monitoring of EC ratios.

**Fig 2:**
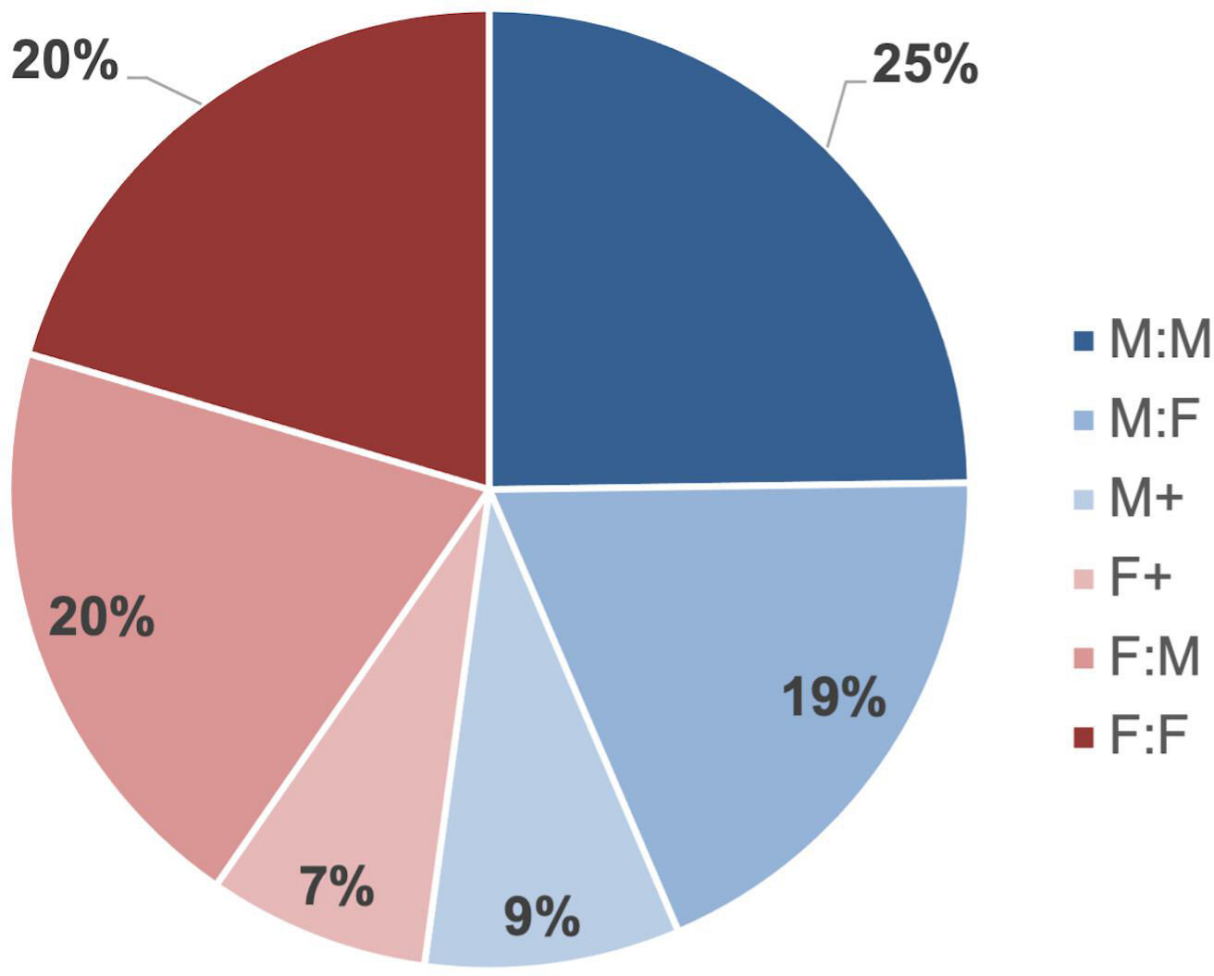
Proportion of gender combinations among EC authors in all ASM journals between 2019 and 2021. M:M, M:F, F:M, and F:F represent papers in which two authors share a position, with the order of authors being male-male, male-female, female-male, and female-female, respectively. For papers with three or more authors sharing an EC position, “M+” means that the first listed author is a male, and “F+” means that the first listed author is a female. The proportion of combinations in which a male author is listed first is shown in various shades of blue, while female-first combinations are shown in shades of red.

When viewing data from all 3 years of entries, a male author was listed first 53% of the time (Fig. 2). While 20% of entries were female-male, 19% of entries were male-female. These data suggest that, at least in ASM journals, gender bias among EC author order was minimal between 2019 and 2021.

### Given reasons for author order

**Fig 3:**
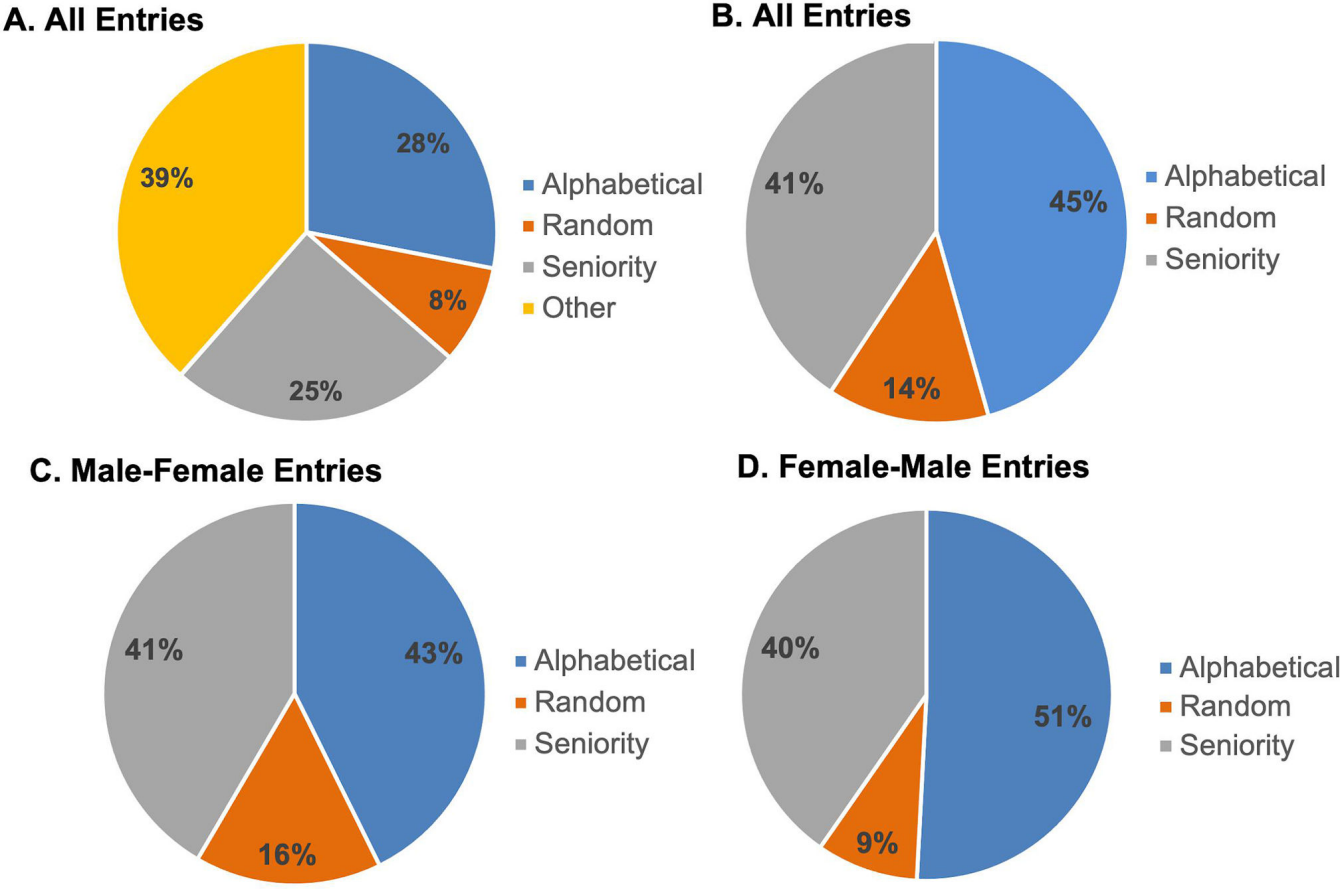
Author-provided justification for ordering EC authors among entries which provided an answer. (**A**) Percentage of cited methods among alphabetical, random, seniority, or other categories. Some entries listed more than one method (*n* = 1,561). (**B**) Percentage of cited methods among alphabetical, random, and seniority categories. Some entries listed more than one method and thus are coded into multiple categories (*n* = 960). (**C**) Percentage of cited methods among alphabetical, random, and seniority categories among entries with a male-female EC byline (*n* = 178). (**D**) Percentage of cited methods among alphabetical, random, and seniority categories among entries with a female-male EC byline (*n* = 181).

Corresponding authors gave a variety of reasons for their EC author order, with most entries being ordered alphabetically, randomly (such as by a coin toss or drawing straws), or by seniority (Fig. 3A). Among these three methods, alphabetical order was the most commonly used method (Fig. 3B). Even among these methods, authors diverged in their use of forward or reverse alphabetical order and forward or reverse seniority. With regard to the methods used for ordering authors, the two-author EC entries that listed a female first were significantly less likely than male-first entries to employ chance as an ordering mechanism (*P* = 0.047).

A significant portion of entries used other methods such as mutual agreement, which collaborator started or finished the project, or which collaborator took on most manuscript-writing responsibilities as opposed to raw research (or vice versa). For 163 entries, there was no reason for EC author order, which may reflect a lack of clarity in submission instructions or a lack of journal enforcement of this requirement.

Several entries gave more unique explanations for how they determine EC author order and were also categorized as “other.” These explanations to include a video game (12), first name length (13), or a collaborator’s personal connection to the manuscript topic (14). One entry explicitly stated that it prioritized the female collaborator (15). Multiple entries stated that they based their order on stroke count of the author’s Chinese names (16–20), a practice used in China to organize names in a uniform order.

**Fig 4:**
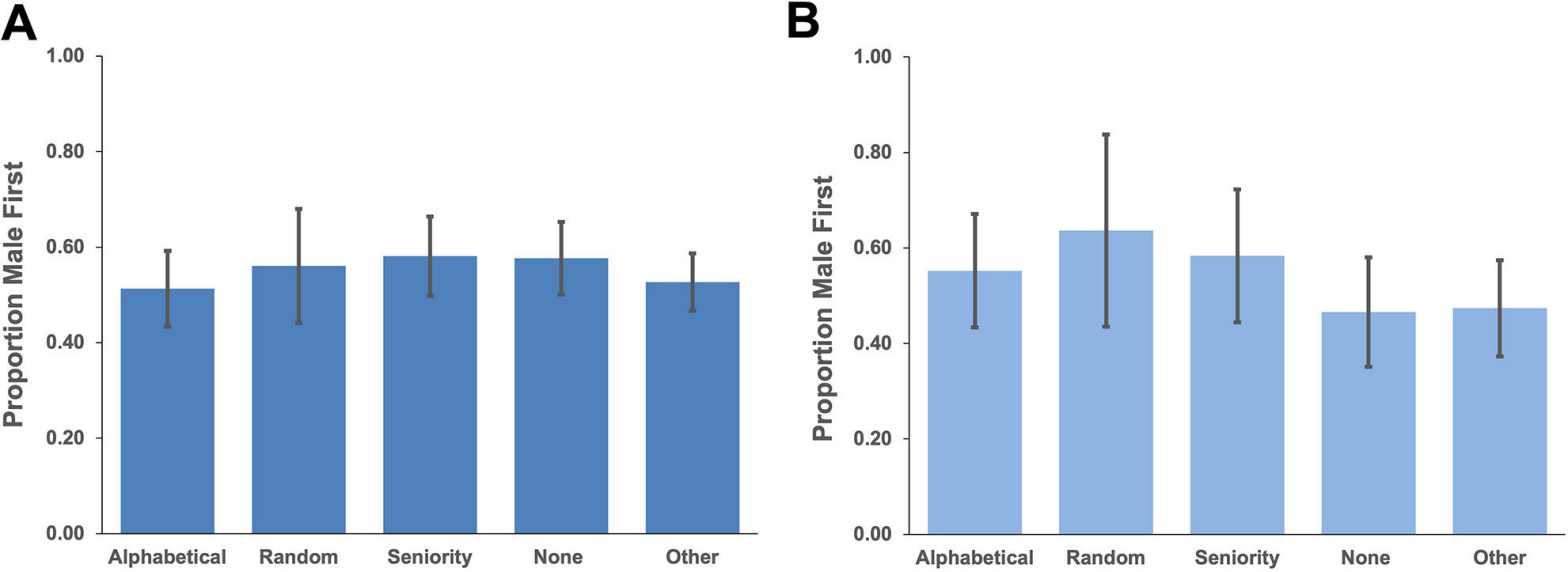
Gender bias in EC entries organized by method used to determine author order. Bars represent 95% confidence interval. Entries that cited multiple methods or that had multiple EC author positions were excluded. (**A**) Percentage of all EC entries with a male first author (*n* = 719). (**B**) Percentage of two-author gender-discordant EC entries that list the male collaborator first (*n* = 305).

From these methods, the research team investigated whether certain methods were associated with a higher likelihood of gender bias. For example, since women tend to occupy fewer STEM leadership positions (9), they may be less likely to be listed first if a lab orders authors based on seniority. While all methods resulted in a male gender bias close to 50%, male authors were more likely to be listed first among all EC categories either if seniority was used to order authors or if an explanation for EC order was not provided (Fig. 4A and B).

In past studies, researchers have suggested that using mutual agreement to determine author order disadvantages women, whom may be less likely to negotiate (21) or promote their accomplishments (22). Our data had 21 entries that listed the phrase “mutual agreement” in their explanation, 11 of which listed a male author first and 9 of which listed a female author first. This sample is too small to support or refuse the above hypothesis.

### Regional variations in results

**Fig 5:**
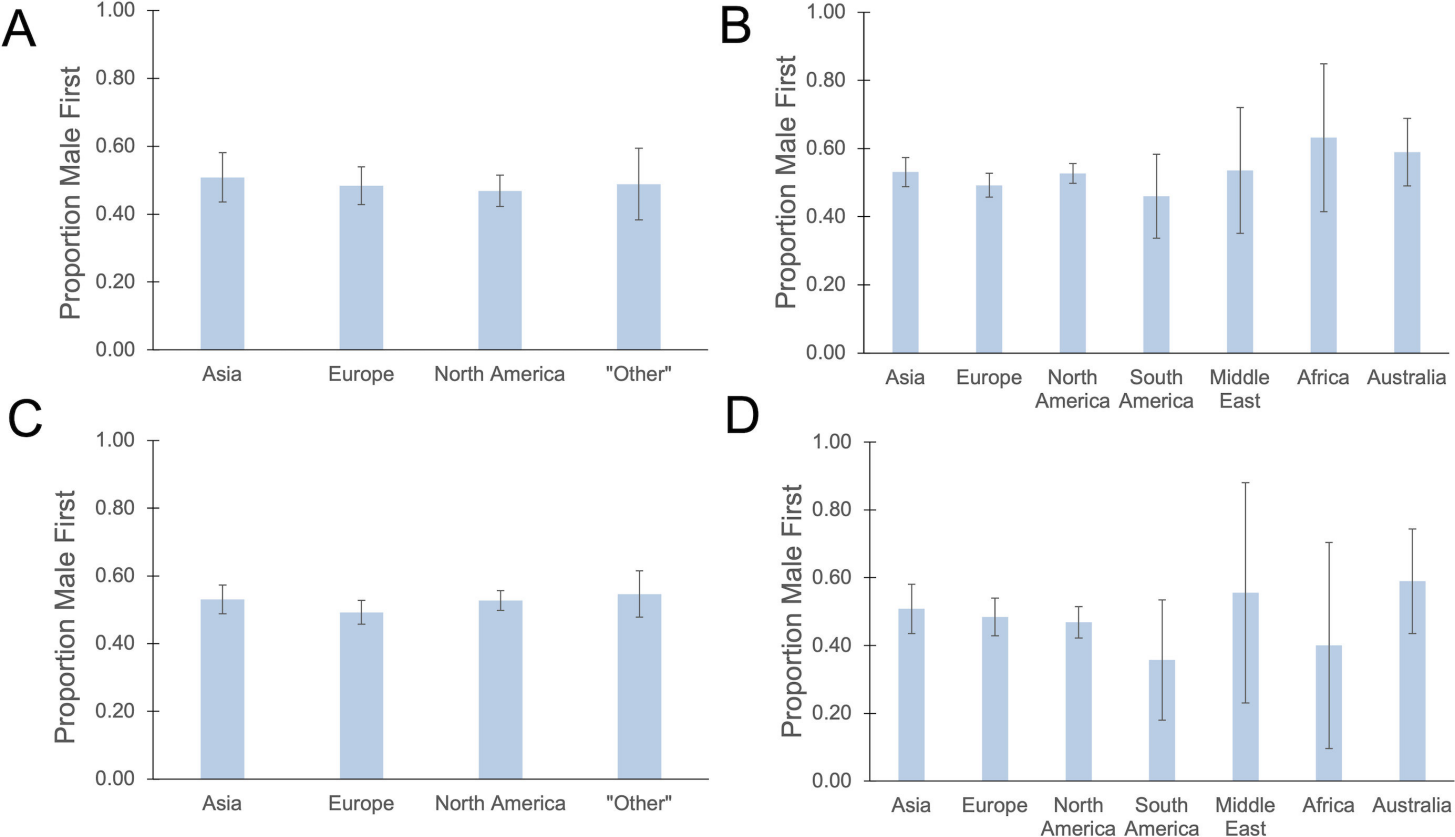
Male gender bias among EC authors organized by world region. Percentages closer to 50% are assumed to have less overall gender bias. Bars represent 95% confidence interval. Publications that were the result of inter-continental collaborations were excluded, and one publication can have multiple EC positions. (**A**) Percentage of all EC publications in which a male author is listed first by world region (*n* = 2,629). (**B**) Percentage of two-author gender-discordant EC publications in which a male is listed first (*n* = 1,027). (**C**) Percentage of all EC publications in which a male author is listed first by Europe, North America, Asia, and other regions (*n* = 2,629). (**D**) Percentage of two-author gender-discordant EC publications in which a male is listed first (*n* = 1,027).

The publications our team analyzed came from research groups based in seven different world regions: North America, South America, Europe, Africa, the Middle East, Asia, and Australia. The majority of entries came from North America, Europe, and Asia. Gender bias did not differ significantly between world regions (Fig. 5A through D).

**Fig 6:**
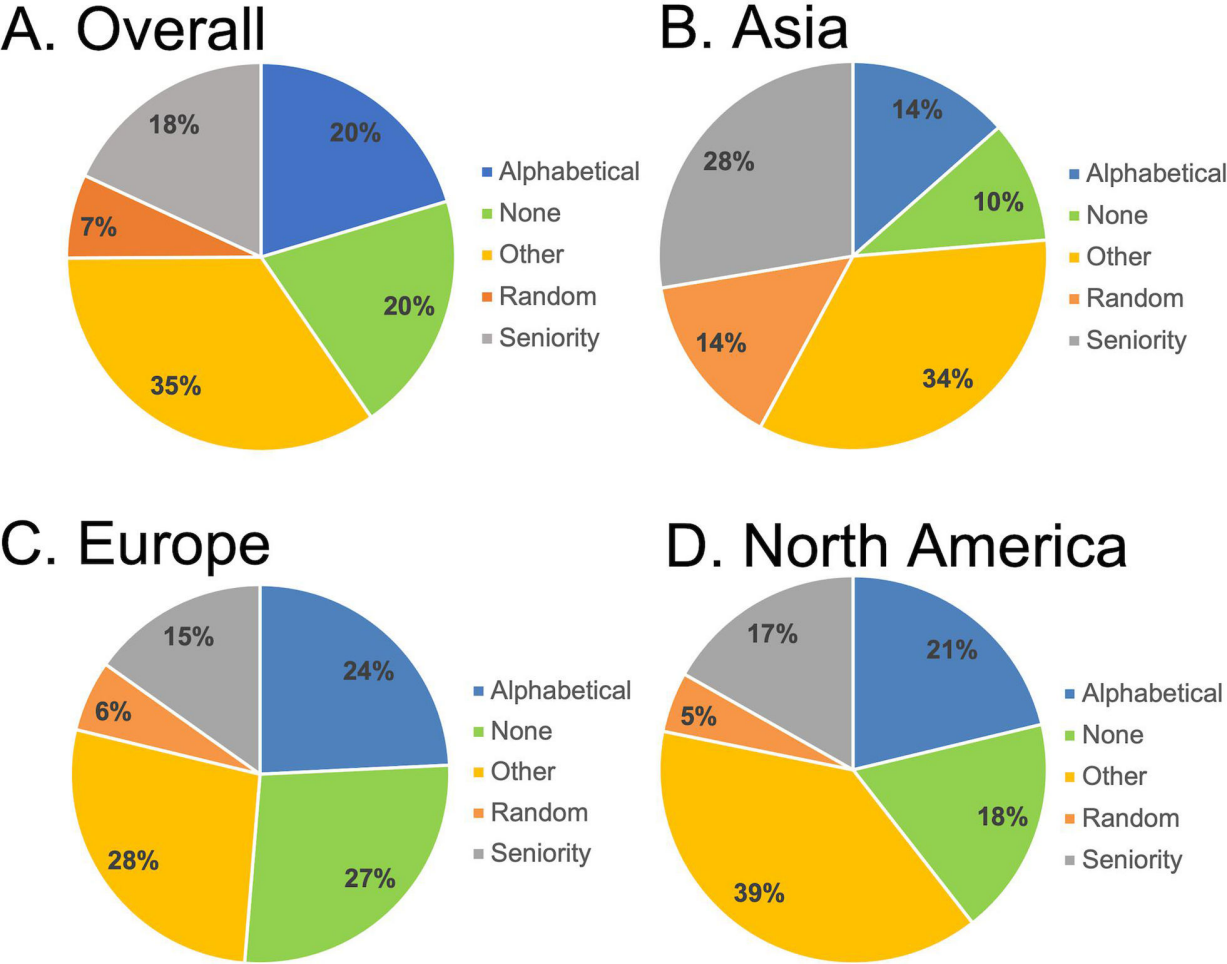
Distribution of EC ordering methods by Europe, Asia, and North America. Publications that were the result of inter-continental collaborations or that cited multiple methods of ordering authors were excluded. (**A**) Distribution of methods in all three regions, including the “other” or “none” category (*n* = 1,591). (**B**) Distribution of methods used in papers with research teams based in Asia, including the “other” or “none” category (*n* = 304). (**C**) Distribution of methods used in papers with research teams based in Europe, including the “other” or “none” category (*n* = 462). (**D**) Distribution of methods used in papers with research teams based in North America, including the “other” or “none” category (*n* = 697).

Among Europe, North America, and Asia, regional patterns emerged in terms of how EC authors were ordered (Fig. 6). For example, research teams in Asia were significantly less likely to alphabetize their authors (*P* < 0.00001) and more likely to use either seniority (*P* = 0.002) or chance (*P* = 0.00002) compared to European and North American research teams. When comparing methods between European and North American research teams, no statistically significant differences were determined.

## DISCUSSION

In this study, we analyzed the gender distribution and byline order of EC authors before and after ASM began requiring corresponding authors to explain how bylines were determined. Past studies have shown that male authors were in some instances more likely to be placed first in an EC byline due to the prestige associated with being a first author (6). Although this pattern of gender bias has been on the decline (6), determining more recent EC byline practices among research groups is important in assessing the best policies to ensure scientists receive fair attribution for their work. In this regard, we noted a slight but noticeable increase in publications with males in the first-author position after the requirement of written explanations in 2020. Although this difference did not reach statistical significance (*χ*^2^ *P* = 0.012), it supports several other studies that note a general decline in female-first authorship across scientific fields in 2020 due to the COVID-19 pandemic (23, 24). The pandemic disproportionately affected female scientists due to difficulties securing childcare (23), and it is possible that our observed trend was a product of this phenomenon. Nevertheless, continued monitoring is warranted as it would be unfortunate if a requirement intended to increase fairness inadvertently introduced a new bias against women. Overall, data on how research teams determine EC order provide an important glimpse into the understudied diversity of laboratory social dynamics (25).

Every EC author pair, no matter the research group a publication comes from, is presented the same way at the top of a journal article. However, every author pair is different, and different laboratories appear to use different methods for determining which author is placed first in an EC pair. The results show that while most research groups use some combination of chance, alphabetization, or seniority to determine author order, even these methods are hardly a monolith. For example, some laboratories might use seniority to prioritize an author who has been in the lab for longer, while other laboratories might use reverse seniority to ensure an untenured author gets a more prestigious entry on their CV. Some laboratories base their decisions on other factors like who writes a manuscript, who finishes a project, or even who wins a game. This diversity highlights the importance of providing an EC statement so readers can have an accurate picture of how the author order was determined.

Specific methods of ordering EC authors often had a close to 50% chance of listing either a man or a woman first. However, our analysis did find that male-female entries were significantly more likely than female-male entries to employ chance. It is difficult to interpret this finding, as chance is often thought of as the method that would result in the least amount of bias. It is possible that, as less than 10% of all the entries analyzed employed chance, this phenomenon would not appear in a larger sample size.

Overall, most world regions display a diversity of methods to determine author order, but research teams based in Asia are less likely to use alphabetical order for their EC authors. One possible explanation for this finding is that research teams in Asia are more likely to use native writing systems rather than English or a Western language amenable for name ordering using the Latin alphabet. Indeed, several research groups based in China reported using stroke count of the researchers’ Chinese names to properly organize them. Given the increasing international nature of scientific publishing, journals should be conscious of this and other cultural differences between global research teams when recommending more “objective” methods of standardizing author order.

This study is not without limitations. To start, the data were analyzed using a man/woman binary and did not collect data on researchers who identify as nonbinary. Requesting voluntary gender identity information upon submission or employing a larger sample size could help determine if these researchers experience gender as in EC deliberations. Additionally, an author’s gender was often identified based solely on the author’s name. While further investigation was conducted for authors with names that were determined to be gender-neutral, there is a possibility that authors with seemingly gender-specific names could have been misidentified.

The data set was limited to published articles in a collection of journals focused on microbiology and allied sciences, so we do not know its applicability to other fields. Alphabetical ordering is a much more established convention in the fields of mathematics, economics, physics, and business, so results might skew toward this method if this study were replicated in these fields (26). In addition, even when some authors were asked to elaborate on how they determined byline order, 9% of published papers did not include an explanation. This may reflect a lack of clarity in the submission instructions, which could be improved in future analyses through the enforcement of journal requirements.

Even if bias for placing male EC authors first has largely declined, its legacy likely remains, particularly when it comes to the CVs of senior female scientists who began their careers when this bias was more prominent. Getting many first-author publications is seen as important for establishing young scientists’ careers, and this pattern could contribute to current inequities such as the fact that women make up 60.81% of research staff but 34.82% of faculty members (8). More research is needed in this area to determine what the legacy of gender bias from previous decades has on women’s careers.
